# Subtotal Resection of an Anaplastic Ganglioglioma in Pregnancy

**DOI:** 10.1155/2018/4606354

**Published:** 2018-05-10

**Authors:** Matthew J. Bicocca, Andrea R. Gilbert, Saeed S. Sadrameli, Michael L. Pirics

**Affiliations:** ^1^Department of Obstetrics and Gynecology, Houston Methodist Hospital, Houston, TX, USA; ^2^Department of Pathology and Genomic Medicine, Houston Methodist Hospital, Houston, TX, USA; ^3^Department of Neurosurgery, Houston Methodist Hospital, Houston, TX, USA; ^4^Department of Obstetrics and Gynecology, Weill Cornell Medical College, New York, NY, USA

## Abstract

**Background:**

Anaplastic ganglioglioma is a rare malignant brain tumor associated with high morbidity and mortality. The diagnosis of a central nervous system malignancy in the early 3rd trimester presents management challenges to both neurosurgeons and obstetricians.

**Case:**

A 33-year-old woman, gravida 2 para 1, presented at 28 6/7 weeks with four months of worsening headaches, nausea, vomiting, and mental status changes due to a 7.5 cm anaplastic ganglioglioma. Maternal deterioration necessitated subtotal tumor debulking allowing prolongation of the gestation to 34 6/7 weeks. After delivery, the patient underwent further resection, followed by chemotherapy and radiation. Both mother and infant are well.

**Discussion:**

This case underscores the importance of timely diagnostic imaging in pregnant women and demonstrates subtotal tumor debulking as a viable means of prolonging gestation.

## 1. Introduction

Primary brain tumors in pregnancy are a rare cause of maternal morbidity with an estimated incidence of 3.6 malignant brain tumors per one million live births [1]. Although the overall incidence of primary intracranial neoplasms in pregnant women is slightly decreased compared to age matched nonpregnant counterparts [1], the dramatic physiologic and hormonal changes of pregnancy are associated with a more complicated clinical course, exacerbated maternal symptoms, and increased velocity of diametric tumor expansion [2, 3]. Additionally, pregnant patients with malignant primary brain tumors have elevated rates of fetal growth restriction, maternal mortality, hyperemesis gravidarum, and cesarean delivery [4].

Ganglioglioma (GG), a rare glial-neuronal brain tumor characterized by dysplastic ganglion cells and neoplastic glial cells, accounts for about 0.4% of all tumors arising in the central nervous system (CNS) [5]. The malignant subtype of GG, called anaplastic ganglioglioma (AGG), comprises about 1–8% of all adult and pediatric GGs and has an estimated incidence of 0.02 cases per million per year [6]. Although there are reported instances of a lower grade GG arising in a pregnant woman [7] and an AGG diagnosed during the postpartum period [8, 9], we are the first to document a case of an AGG diagnosed during pregnancy.

## 2. Case Presentation

The patient was a 33-year-old woman, gravida 2 para 1, with an intrauterine pregnancy dated by a 9-week ultrasound. She had an obstetrical history of one prior vaginal term delivery and a family history of breast cancer. Beginning at 11 weeks gestation, she reported headaches that were not relieved by acetaminophen, as well as a pulsing sensation in her left ear. Treatment was initiated with acetaminophen/butalbital/caffeine (Fioricet) for presumed migraine headaches. At 14 weeks, she complained of new onset jaw-tightness and a vibrating sensation in her ears, which prompted a referral to a primary care physician. At 20 weeks, the fetal anatomy scan showed appropriate growth with no fetal anomalies, but the patient's headaches had worsened and required frequent narcotic use. Four weeks later, the patient was referred to a neurologist who attributed her worsening symptoms to occipital neuralgia. Beginning at 26 weeks, her headaches were accompanied by anorexia, nausea, and vomiting. She also experienced two vasovagal episodes with questionable seizure activity. Psychiatric changes, which included severe depression and neglected hygiene, prompted two visits to the emergency department where she was diagnosed with migraine headaches and received opiates and antiemetics.

**Figure 1:**
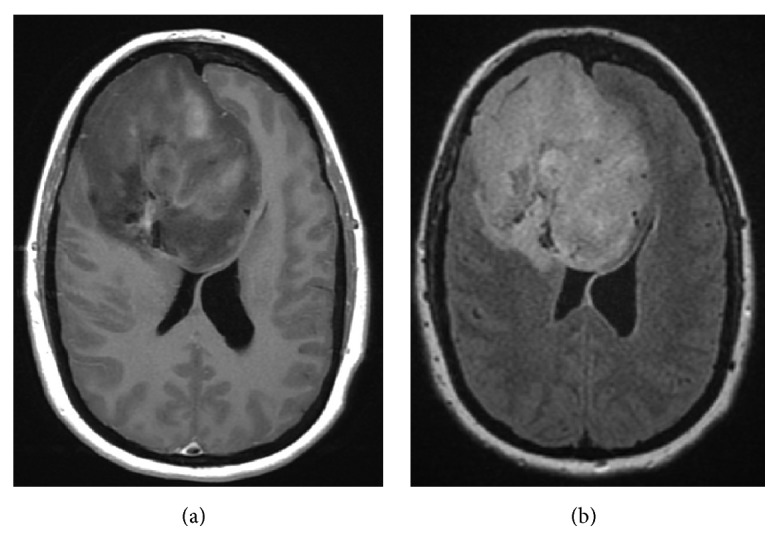
Axial MRI shows a 7.5 cm heterogenous mass in the right frontal lobe with T1-weighted sequences showing patchy enhancement following contrast administration (a) and hyperintensity with surrounding vasogenic edema on T2-weighted fluid attenuated inversion recovery sequences (b).

At 28 6/7 weeks, the patient underwent a workup for preeclampsia for which laboratory investigations were negative. However, magnetic resonance imaging (MRI) of the brain revealed a 7.5 cm heterogenous mass in the right frontal lobe (Figure 1) that showed patchy contrast enhancement, focal calcifications with blood products, and surrounding vasogenic edema with mild midline shift. At this time, the patient was transferred to our institution for the remainder of her care.

On admission, she was noted to have sluggishly reactive pupils and mild weakness in the left upper extremity. An ultrasound confirmed appropriate estimated fetal weight and amniotic fluid level. Levetiracetam was administered as well as dexamethasone, which was switched to methylprednisolone after 48 hours.

Three days following transfer of care, the patient showed evidence of clinical deterioration, including recurrent aphasia and a generalized atonic seizure. The decision was made to proceed with neurosurgical resection of the brain mass to relieve intracranial pressure. Given the extent of the mass, involvement of eloquent cortex, and safety concerns for the fetus and the mother, the operation was planned in a staged fashion, with the second stage scheduled after delivery. At 29 4/7 weeks, the patient underwent a bifrontal craniotomy and subtotal right frontal lobectomy with excision of a highly vascular tumor; the posterior aspect of the neoplasm encroached on the motor cortex and was not resected at that time. Estimated blood loss was 100 mL. Continuous fetal heart monitoring performed throughout the operation showed a baseline of 135 beats per minute with minimal variability and no acceleration or deceleration. Once in recovery, the fetal heart tracing improved to moderate variability with 10 × 10 acceleration. Postoperatively, the patient recovered well. Her neurological status improved with resolution of her aphasia and cessation of seizure activity. She was discharged home on postoperative day two.

**Figure 2:**
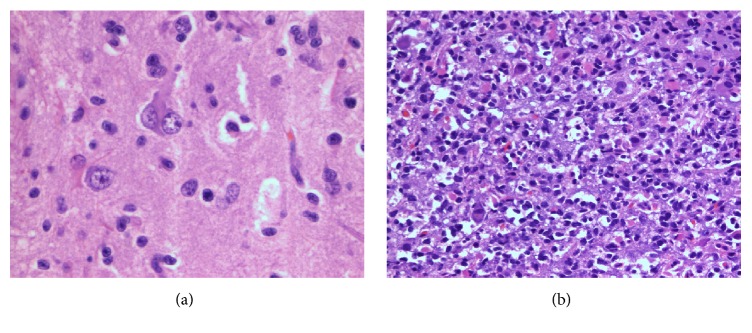
H&E stain shows a biphasic neoplasm characterized by dysplastic ganglion cells, including binucleate forms ((a) 400x magnification), and a hypercellular malignant glial component with nuclear atypia and increased mitotic activity ((b) 200x magnification).

Hematoxylin and eosin (H&E) staining and immunohistochemistry were performed on sections prepared from formalin-fixed paraffin-embedded tissue. Microscopic examination showed hypercellular neuroglial tissue featuring GFAP-positive neoplastic astrocytes and clusters of dysplastic-appearing ganglion cells highlighted by NeuN and synaptophysin immunostains. The ganglioglioma was qualified as anaplastic due to increased cellularity, nuclear atypia, and mitotic activity in the glial component (Figure 2).

Due to the highly aggressive nature of AGG, a compromise was reached between prolonging gestation and delaying further treatment. At 34 6/7 weeks, a viable 2800 gram male infant was born via cesarean delivery with Apgar scores of 7 and 8 at one and five minutes, respectively. Eleven weeks after the initial resection and five weeks after delivery, the patient underwent the second operative phase consisting of an awake-craniotomy with neuronavigation. There was complete gross resection of residual tumor, except for a small portion attached to the anterior cerebral artery.

Intensity modulated radiotherapy to the brain was initiated two weeks after the operation. The patient underwent 2 Gy external beam radiotherapy in thirty sessions for a total of 60 Gy. She then completed twelve cycles of adjuvant temozolomide with excellent tolerability. The patient is now two years status post-initial tumor resection. She has no neurologic sequelae; however, her latest MRI showed continued slow growth of an enhancing 1.7 cm left frontal calvarium lesion. The clinical significance is yet undetermined. Her infant required hospitalization in the neonatal intensive care unit for 19 days due to feeding difficulties prior to discharge but is currently doing well.

## 3. Discussion

Headache is a very common patient complaint, but new onset or worsening headaches in a pregnant woman should raise concern. A broad differential must be considered and include common etiologies, such as migraines, tension, or cluster type headaches, as well as pregnancy specific causes like preeclampsia. Other rarer and often grave etiologies should also be entertained, including intracranial hemorrhage, cerebral venous sinus thrombosis, infectious meningitis/encephalitis, and brain tumors.

Glial tumors diagnosed in pregnancy are rare, mostly being reported in case series or case reports, and have a decreased incidence compared to age matched nonpregnant counterparts [1]. Yust-Katz et al. performed a retrospective review at The MD Anderson Cancer Center over seventeen years and found only ten patients diagnosed with gliomas while pregnant and an additional five diagnosed in the immediate postpartum period, none of whom had AGG [10]. A 2017 case series reviewed 75 cases of pregnant women in whom new onset glioma developed: 42 women had high grade gliomas, none with AGG, and there were seven maternal deaths. They note that all types of antitumor therapy, including surgical resection, chemotherapy, and radiation, have been safely applied during pregnancy, but consideration must be given to the gestational age and maternal symptoms at time of presentation [11].

GG is a rare glial-neuronal brain tumor characterized by neoplastic glial cells and dysplastic neuronal cells. According to the recently revised fourth edition of the World Health Organization (WHO) Classification of Tumors of the CNS, GG is considered to be WHO Grade I neoplasm [5]. The criteria for Grade II designation are not well defined, but tumors that demonstrate overt malignant features like increased cellularity, nuclear atypia, and mitotic activity, typically in the glial component, are classified as a Grade III AGG [5].

There is a single report of a woman diagnosed with a WHO Grade I GG prior to becoming pregnant who had accelerated tumor growth during pregnancy [3]. Similar findings have been reported in women harboring gliomas, including one study, gathered from a multi-institutional database, of eleven women with Grade II diffuse gliomas who experienced significant increase in the velocity of diametric tumor expansion during pregnancy [2]. Complications secondary to accelerated tumor growth, compounded by dramatic physiologic changes of pregnancy, increase the risk of adverse outcomes and underscore the importance of timely diagnosis [4]. Pregnancy should not delay appropriate diagnostic testing in the workup of patients with headache nor should it delay management of pregnant patients diagnosed with a brain tumor.

To our knowledge, the English literature has only one report of a low grade GG initially diagnosed during pregnancy [7] and two cases of AGG diagnosed in the postpartum period [8, 9], but our patient is the first report of an AGG diagnosed during pregnancy. The patient presented with headaches, a common complaint with a broad differential. This case underscores the importance of timely diagnostic imaging in pregnant women and demonstrates subtotal tumor debulking as a viable means of prolonging gestation. As was the case in our patient, a collaborative approach to management of brain tumors in pregnancy should involve multiple specialties, such as maternal-fetal medicine, neurosurgery, and obstetrics, to optimize patient care and minimize risks to the fetus and mother.
